# Dietary macronutrient content and energy intake in the mouse: hedonic or homeostatic override?

**DOI:** 10.1002/oby.24312

**Published:** 2025-06-04

**Authors:** Stephen J. Simpson, Alistair M. Senior, Samantha M. Solon‐Biet, David G. Le Couteur, David Raubenheimer

**Affiliations:** ^1^ Charles Perkins Centre The University of Sydney Sydney Australia; ^2^ School of Life and Environmental Sciences, Faculty of Science The University of Sydney Sydney Australia; ^3^ Faculty of Medicine and Health The University of Sydney Sydney Australia; ^4^ Ageing and Alzheimer's Institute and Centre for Education and Research on Ageing Concord Hospital Concord Australia

## Abstract

**Objective:**

The objective of this study was to reconcile how two landmark mouse studies came to opposite conclusions regarding the relationship between dietary macronutrient composition and energy intake. Hu et al. concluded that dietary fat drives excess energy intake because its hedonic properties override energy homeostasis. Solon‐Biet et al. concluded that energy intake increases with dietary fat owing to the dilution of protein and carbohydrates, with compensatory feeding for these nutrients dominating inhibitory feedback from fat.

**Methods:**

Nutritional geometry was used to reanalyze data from Solon‐Biet et al. and Hu et al.

**Results:**

Results from the two studies are strongly concordant. Neither was designed to measure hedonics but, in both studies, the positive associations among dietary fat, food, and energy intakes are as predicted by compensatory feeding for dietary protein and carbohydrates without the need to impute hedonic effects of fat.

**Conclusions:**

Whereas conclusions cannot be drawn from either study regarding the role of hedonics, there is evidence for homeostatic feedback operating in both. We suggest that hedonic and homeostatic mechanisms likely interact, with homeostasis being more influential over the longer term. Therefore, “hedonic diversion” may be a more appropriate concept than “hedonic override” when considering energy consumption in mice and, perhaps, humans.

**Study Importance**

**What is already known?**

- Macronutrients influence food and energy intakes both by impacting the sensory qualities of foods (hedonics) and through regulatory feedback (homeostasis).

**What does this study add?**

- We use nutritional geometry to reconcile how two landmark studies came to opposite conclusions regarding the relationship between dietary macronutrient composition and food and energy intakes in mice. Homeostatic feedback could account for the long‐term patterns of energy intake in both studies, but hedonics alone could not.

**How might these results change the direction of research?**

- The study highlights the need to consider how hedonic and homeostatic mechanisms interact across different time scales, with hedonics transiently diverting homeostasis rather than overriding it in the longer‐term control of energy intake.

## INTRODUCTION

Macronutrient composition impacts food intake, both in the short and long term. Effects are mediated by interacting processes that include hedonics, comprising sensory responses to foods and activation of brain reward circuitry, and homeostatic systems, involving, inter alia, circulating metabolites, hormones, and neural feedback signals [1, 2, 3, 4, 5, 6]. Sweet, fatty, salty, and savory flavor characteristics are linked to the hedonic effects of foods and have been implicated in the susceptibility to overconsume industrially processed foods and beverages [7, 8]. Regarding homeostatic processes, mechanisms controlling the intake of specific macronutrients (and some micronutrients) have increasingly become a focus in appetite research, moving away from a concentration on undifferentiated energy intake [3, 9, 10, 11].

According to the strength of regulatory feedback arising from different nutrients, ingestion of some may be prioritized over others in the control of food intake. An example of nutrient prioritization is the case in which dilution of dietary protein by fiber or other macronutrients results in increased food intake, such that protein “leverages” excess intake of other food components [12, 13, 14]. Protein leverage has been shown to differing degrees in a wide range of species, ranging from near complete protein prioritization in some primates [15] to some predatory species that show stronger leveraging of food intake by fats than by protein [16, 17], as predicted by theory from trophic and nutritional ecology [3, 18]. The protein leverage hypothesis proposes that protein leverage in humans has interacted with changes in the modern industrialized food supply, by which protein has become diluted by carbohydrates and fats to affect energy overconsumption and obesity [12, 13, 14].

The mouse has been an important system for studying the interactions among macronutrients in controlling food and energy intakes, as well as the associated consequences for obesity and cardiometabolic health [19, 20, 21]. There is evidence that mice possess separate appetite systems for protein and carbohydrates, which compete under restriction to a fixed diet; when offered choices among nutritionally complementary foods, mice select and regulate to a ratio of these nutrients containing ~25% energy as protein [22]. Similar findings are reported in rats [23].

In the past decade, two landmark studies that systematically explored the relationship between macronutrient mixtures and food intake in mice have arrived at seemingly contrasting conclusions regarding the relative roles of hedonics and nutrient‐specific regulatory feedback on food intake.

Solon‐Biet et al. [2] quantified the interactive effects of dietary protein, carbohydrates, fats, and energy density on food intake in male and female C57BL/6 mice maintained throughout life on 1 of 25 diets. They concluded that “regulatory feeding effects were most evident for dietary protein and less marked for dietary carbohydrate. In contrast, fat content in the diet was largely unregulated and thus had negligible influence on food intake.” Thus, mice increased food intake as protein (and, to a lesser extent, carbohydrate) concentration fell in the diet, with fat ingestion following more passively and essentially acting as a highly energy‐dense diluent of these nutrients. Consequently, the combination of a low‐percent protein, low‐percent carbohydrate, and high‐percent fat diet resulted in the greatest total energy intake.

Hu et al. [1] subsequently used 29 experimental diets to further explore the relative roles of dietary macronutrients on energy intake and body composition in young male mice (C57BL/6 and four other strains). In contrast to Solon‐Biet et al. [2], these authors concluded that “mice regulate their food consumption primarily to meet an energy rather than a protein target, but this system can be over‐ridden by hedonic factors linked to fat, but not sucrose, consumption.” Several studies have now reanalyzed specific subsets of the data from the experiment described by Hu et al. [1] using a suite of different methods. Such studies have reached similar conclusions regarding a primary causal role for dietary fat and hedonic factors in governing excess energy intake, weight gain, and reduced metabolic health [24, 25, 26].

Our aim in the present study was to apply the integrative nutritional geometry framework [19, 27] to examine how the seemingly contradictory results of these two seminal studies [1, 2] might be reconciled.

## METHODS

We used nutritional geometry to reanalyze published and previously unpublished data from the experiment described by Solon‐Biet et al. [2], alongside published data from the study by Hu et al. [1]. Both studies used commercially sourced diets and C57BL/6 mice, although Hu et al. [1] also assessed effects of diet in C3H, DBA2, BABL‐c, and FVB mice; see Solon‐Biet et al. [2] and Hu et al. [1] for details regarding animal and diet sourcing. Animals in the experiment by Hu et al. [1] were single‐housed at 22°C. In Solon‐Biet et al. [2], ethical considerations required that mice were housed in cages of three animals of the same sex and at 25°C.

For Hu et al. [1], data on nutrient and energy intakes were taken from the Supplemental Information. We have confirmed with the authors that there are some miscalculations in tabulated values of macronutrient compositions of experimental diets in Table S1 of Hu et al. (see our Figure S1). In the analyses that follow, we have directly calculated the diet macronutrient percentages from the stated dietary ingredients and applied these revised values to food intake data to calculate macronutrient and energy intakes (Table S1). Our analyses established that the errors are not responsible for the differing interpretation of the two studies. Data from Solon‐Biet et al. [2] were the same as those used in that publication and were calculated based on food intake averaged over the period between ages 6 and 15 months. Hu et al. [1] used young male mice (the study ran for 3 months, from age 10 weeks). Herein we also present, for the first time, data at 24 weeks from Solon‐Biet et al. [2].

**FIGURE 1:**
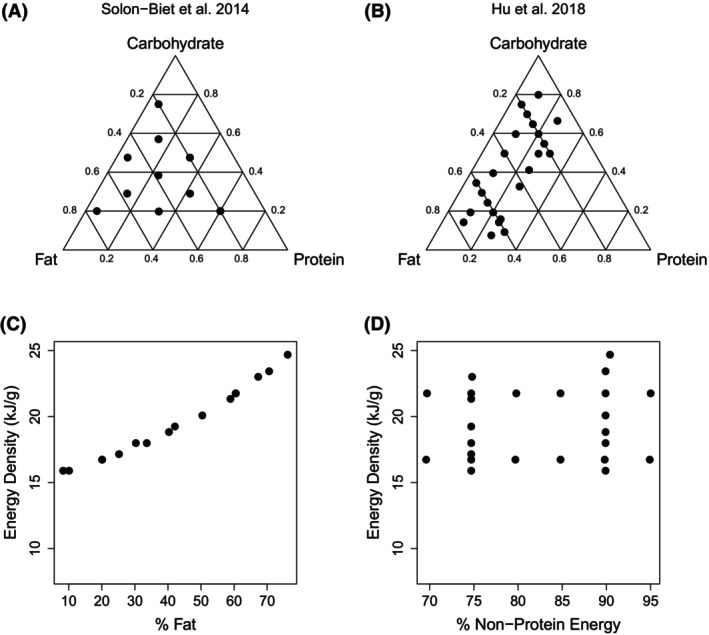
Comparison of dietary macronutrient compositions. (A) Composition of diets used in Solon‐Biet et al. [2] in terms of proportion of energy from protein, carbohydrate, and fat. Note that each composition was tested at three energy densities (i.e., 8 kJ/g, 13 kJ/g, and 17 kJ/g) achieved by dilution with nondigestible cellulose. (B) Composition of diets used in Hu et al. [1]. Axes as in panel A. (C) Percentage of dietary energy from fat, against dietary energy density (kilojoules per gram), and (D) percentage of nonprotein energy (carbohydrate + fat) against energy density in the diets used by Hu et al. [1]. See also Table S1.

Solon‐Biet et al. [2] used 10 macronutrient ratios, which sampled three‐way combinations of protein (5%–60% of total energy), fat (20%–75%), and carbohydrate (20%–75%; Figure 1A and Table S2). Each of these compositions was provided at a total energy density of 8, 13, or 17 kJ/g by addition of cellulose. This design enabled the interactions among protein, carbohydrate, and fat content on response variables (life‐span, intakes, cardiometabolic phenotype, etc.) to be statistically modeled, together with the impact of dietary energy density/cellulose content. Covariance among variables is inevitable in dietary experiments in which the concentrations of focal components are manipulated, owing to the properties of mixtures. Herein, because energy density and cellulose content covaried very strongly (cellulose diluted energy density), these two factors cannot be distinguished readily.

Hu et al. [1] used 29 diets comprising four series (Figure 1B); Hu et al. [1] also included a series testing for the effects of sucrose, although these are not reanalyzed herein (but see the *Discussion* section for details). Series 1 and 2 spanned 5% to 30% protein (i.e., a lower upper limit for percent protein than in Solon‐Biet et al. [2]), fixing fat at 60% or 20%, with the remainder of energy being digestible carbohydrates; series 3 and 4 tested 10% to 80% fats at either 10% or 25% protein, with carbohydrate again making up the remainder. Hu et al. [1] presented their data for the different diet series separately, plotting food and energy intakes against protein content (series 1 and 2) and fat content (series 3 and 4). The interactions among the three macronutrients were not fully explored, nor was the impact of energy density.

It is routine in nutritional geometry to explore the three‐way effects of the macronutrients using general additive models (GAMs) interpreted via nutrient response surfaces (e.g., 2). One challenge in doing so for the data of Hu et al. [1] is that, in their design, the fat content and energy density of the diets covaried, with the correlation approaching unity (Figure 1C). This arises because fat is approximately double the energy density of protein and carbohydrate. Accordingly, for the same reason that Solon‐Biet et al. [2] could not readily distinguish dietary energy density from cellulose content in their modeling, it was not possible to separate the effects of fat as a nutrient from the effects of total energy density in the Hu et al. [1] data. This feature of the experimental design precluded a three‐dimensional GAM analysis of the effects of protein, fat, and carbohydrate, as was undertaken in Solon‐Biet et al. [2]. In contrast, total nonprotein energy was uncorrelated with total energy (Figure 1D). It was therefore possible to use a two‐dimensional analysis testing the effects of dietary concentration of protein versus nonprotein energy (i.e., fat and carbohydrate combined as a single nonprotein energy axis) on food intake. Although a single nonprotein axis cannot disentangle the individual contributions of fat and carbohydrate, the test of strength of appetite responses for fat and carbohydrate combined relative to dietary protein is robust.

We used two‐dimensional GAMs of the following form:

(1) $$y_{i}=\beta_{0}+f\left(x_{i},z_{i}\right)+\varepsilon_{i}$$

(2) $$\varepsilon_{i}\sim N\left(0,\sigma^{2}\right)$$

where *y*_*i*_ is the *i*th observation of intake (food in grams or energy in kilojoules, depending on the outcome of interest), with dietary protein *x*_*i*_ in kilojoules per gram and nonprotein *z*_*i*_ in kilojoules per gram; *β*_*0*_ is a constant giving expected intake on a diet with the mean protein and nonprotein content of all diets in the study (similar to the intercept in a linear regression where the predictor has been mean‐centered); *f* is a nonparametric smooth function giving the effect of dietary protein and nonprotein on intake; *ε*_*i*_ is the residual for the *i*th data point; and residuals are assumed to be normally distributed with residual variance *σ* ^2^. The terms *β*_*0*_ and *f* are estimated by the model; *β*_*0*_ can be interpreted as the elevation of the nutrient response surface for intake as a function of dietary protein and nonprotein content, whereas *f* gives the shape of the surface. A statistically significant *f* term indicates that intake varies as a function of diet content (i.e., the null hypothesis is a flat surface). GAMs were implemented using the “gam” function in the package *mgcv* [28], specified as “gam(intake ~ s [protein_kcal.g, nonprotein_kcal.g]),” where “s” implements the smooth term as thin‐plate spline.

In order to control for dietary energy density, we performed additional analyses on a subset of 11 near‐isocaloric diets from Hu et al. (ranging from 15.9 to 18 kJ/g in series 2 diets and five lower‐energy diets from series 3 and 4) [1] and analyzed these using linear and nonlinear models (LM and NLM, respectively; being isocaloric precludes a two‐dimensional analysis) alongside the most similar subset of treatments from Solon‐Biet et al. (the 10 macronutrient ratios at ~17 kJ/g) [2].

LMs were implemented as standard linear regression of the following form:

(3) $$y_{i}=\alpha +\beta x_{i}+\varepsilon_{i}$$

where *α* is the intercept, *x*_*i*_ is the density of protein energy in the diet (i.e., from 0 to 1), *β* is the coefficient for the effect of dietary protein on intake, and *y*_*i*_ and *ε*_*i*_ are as described earlier. LMs were implemented using the “lm” function in base R specified as “lm(intake ~ protein)” (R Project for Statistical Computing). Generally, we have analyzed the two datasets separately. However, in places we directly evaluate whether the overall intake or the effect of protein differs between studies by analyzing both studies in a single LM. Differences in overall intake were assessed using LMs specified in base R as “lm(intake ~ study_ID),” where “study_ID” specifies whether data come from Solon‐Biet et al. [2] or Hu et al. [1], and differences in the effect of protein with an interaction between percentage of energy from protein and study identifier specified as “lm(intake ~ protein * study_ID).”

Finally, NLMs were used to test whether intake is affected nonlinearly by the density of dietary protein. Specifically, nutritional geometry predicts that, in order to maintain a constant intake of protein in grams, *P*, on a diet containing a given proportion of protein, p, the animal must eat *P*p^*−1*^ grams of food; for derivation, see Hall [29] and Senior et al. [30]. Therefore, we fitted NLMs of the following form:

(4) $$y_{i}=Px_{i}^{L}+\varepsilon_{i}$$

where *P* is a constant giving the predicted intake on a diet comprising only protein; the strength of leverage is given by the coefficient *L*; and *y*_*i*_, *x*_*i*_, and *ε*_*i*_ are as described in equation 3. The parameters *P* and *L* are estimated by the model; *L* = −1 would be interpreted as complete leverage, *L* = 0 as no leverage, and −1 < *L* < 0 as partial leverage. Note that *L* is equivalent to a regression coefficient measuring the strength of protein leverage and is not a measure of the extent of variation in the data explained by the model. Therefore, even small negative values of *L* could explain large amounts of variation in the data for food and energy intakes depending on the range of protein dilutions tested and the energy content of the dilutant (see *Results* section). NLMs were implemented using the “nls” function in base R, specified as “nls(intake ~ P*protein^L),” which provides a nonparametric *p* value for the *L* coefficient for the null hypothesis *L* = 0.

All statistical analyses were undertaken in the statistical programming environment R version 4.3.1 (R Project for Statistical Computing). All code and data have been made freely available at the following link: https://github.com/AlistairMcNairSenior/Mouse_PL

## RESULTS

### Concordance of results

**FIGURE 2:**
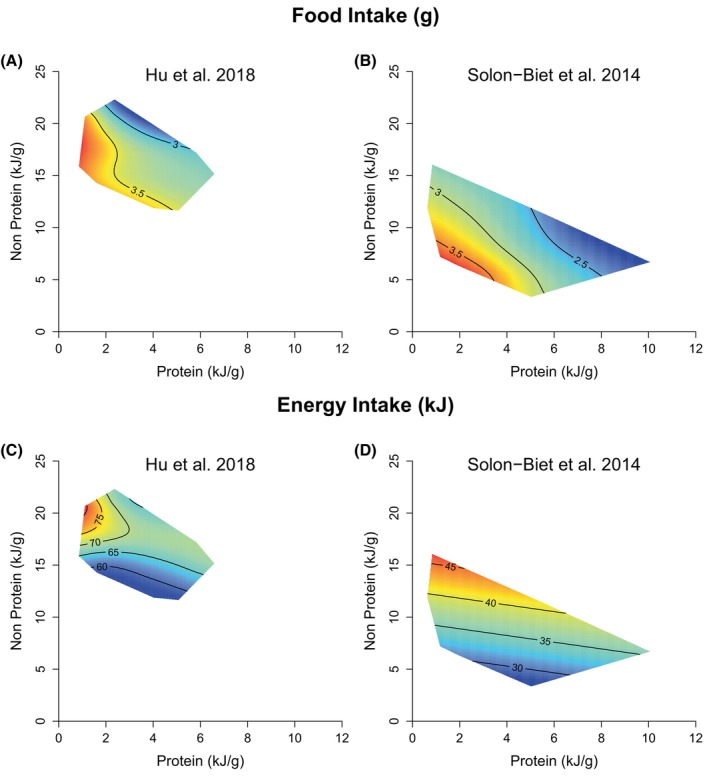
Dietary protein and nonprotein energy influences on food and energy intakes in C57BL/6 mice. Fitted surfaces from GAMs testing for the effects of dietary protein and nonprotein on (A,B) food intake (grams) and (C,D) energy intake (kilojoules) in Hu et al. [1] and Solon‐Biet et al. [2]. For Solon‐Biet et al. [2], GAMs were fitted from data for both sexes, with intakes averaged between 6 and 15 months. In all panels, surface colors are scaled such that maximal values within the panel are given in red and minimal values are given in blue, for any given response. See also Figure S2 and Table S3. GAM, generalized additive model.

Response surfaces for food and energy intakes as a function of dietary protein and nonprotein concentrations for all diets are presented in Figure 2 (see also Table S3). Areas of maximal intake within a study are given in red, and minimal values are in blue. Isolines across each surface indicate the numeric value for each response modeled. There are two conclusions to be drawn. First, the topography of the response surfaces is fundamentally the same in both studies, despite the sampling of different regions of macronutrient space. Food intake was significantly affected both by the protein and nonprotein content of the diet, with these having nonlinear effects. Food intake increased as either protein or nonprotein energy density in the diet fell (Figure 2A,B). In contrast to food intake, total energy intake was highest in diets containing both a low concentration of protein and a high concentration of nonprotein energy (Figure 2C,D). We have reapplied the two‐dimensional GAM approach to each of the different strains of mice tested by Hu et al. [1]. There are some differences in the overall elevation and shape of the response surfaces, indicative of a degree of strain‐specificity (Figure S2). However, in all cases, the individual surfaces show that total energy intake was highest on diets with a low protein concentration and high nonprotein (Figure S2).

The second conclusion to be drawn was that the mice from Hu et al.'s [1] study consistently ate more food and energy across all diets than those in Solon‐Biet et al. [2] (elevation of surfaces in Figure 2). Across the diets as shown in Figure 2, the animals in Solon‐Biet et al. [2] were estimated to eat 28 kJ less per day than those in Hu et al (LM study; estimate [Est.] = −27.97, SE = 0.69, *t* = −40.45, *p* < 0.001) [1]. This difference in energy intake was reflected by differences in body mass and could not be explained by age or sex (Figure S3), leaving the lower housing temperature and/or the single‐housing of animals [31, 32] in Hu et al. [1] as possible explanations for increased energy intake.

### Macronutrient regulation versus hedonic override

The results in Figure 2 and the accompanying GAMs are consistent with macronutrient feedback onto food intake arising from both protein and fat/carbohydrate as distinct from regulation of energy intake per se. In order to further explore macronutrient feedback in the data of Hu et al. [1], we next restricted analysis to the subset of 11 diets (15.9–18 kJ/g) from Hu et al. [1] that were of similar energy density to the 10 high‐energy‐density (17 kJ/g) diets in Solon‐Biet et al. [2].

**FIGURE 3:**
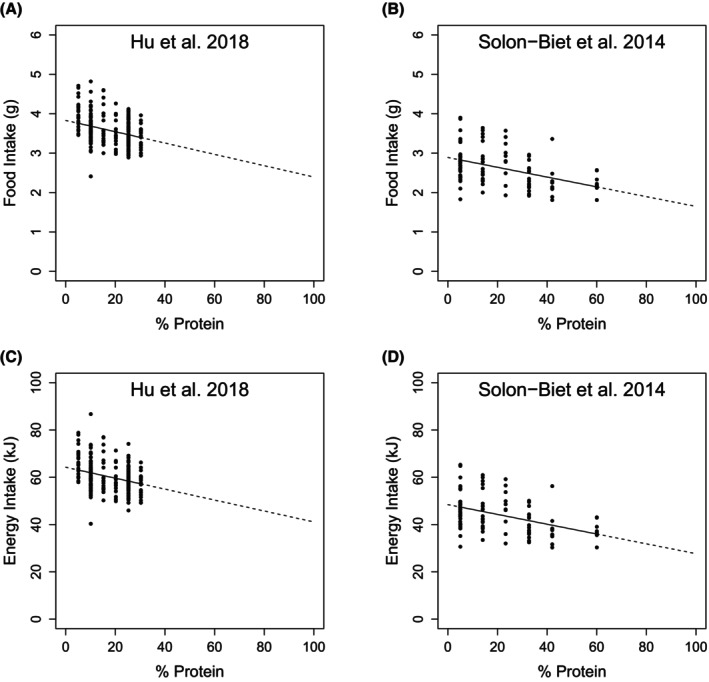
Dietary protein content by energy influences on food and energy intakes. The effect of the percentage of energy from dietary protein on mass of food intake (grams) and energy intake (kilojoules) in C57BL/6 mice. (A,C) Hu et al. [1] and (B,D) Solon‐Biet et al. [2]. Lines correspond to fitted values from linear models (intake = *a* + *b* × percent protein). In Hu et al. [1], data come from diets with between 15.9 and 18 kJ/g to allow more direct comparison with the 17‐kJ/g diets in Solon‐Biet et al. [2]. For Solon‐Biet et al. [2], data are for both sexes, with intakes averaged between 6 and 15 months. See also Table S4.

**FIGURE 4:**
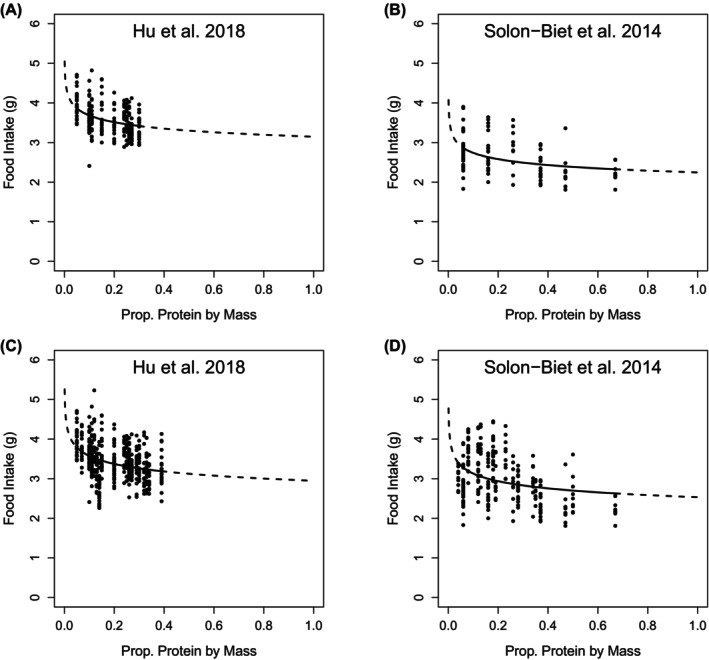
Dietary protein content by mass influences on food intake. Effect of the proportion of protein in the diet by mass on food intake (grams) in both studies. Nonlinear curves correspond to *P*p^*L*^, where *P* is the intake when *p* = 1, *p* is the proportion of the food that is protein, and *L* is the effect of protein leverage. *P* and *L* were estimated from the data. (A,B) Hu et al. [1] data are restricted to between 15.9 and 18 kJ/g and for Solon‐Biet et al. [2] to 17 kJ/g. (C,D) Data from all diets. For Solon‐Biet et al. [2], data are for both sexes, with intakes averaged between 6 and 15 months. For both studies, data are for C57BL/6 mice. See also Table S5.

In this restricted set of approximately isocaloric diets, both food and energy intakes decreased as the percentage of energy from protein increased (Figure 3A–D; see Table S4 for model coefficients). Importantly, this protein leverage effect was of the same magnitude in both studies. Hu et al. [1] did not explore as wide a range of concentrations of dietary protein as Solon‐Biet et al. did [2]. Nevertheless, based on LMs fitting an interaction term between protein density and study identifier, the coefficients for the effect (i.e., slopes) of protein on intake within the two datasets are statistically indistinguishable (LM percent protein × study interaction; food intake [grams], Est. = 0.002, SE = 0.004, *t* = 0.529, *p* = 0.60; energy intake [kilojoules], Est. = 0.024, SE = 0.06, *t* = 0.385, *p* = 0.7). As discussed in the *Methods* section, the correct test of the strength of protein leverage is not an LM but rather a power function. Fitting this model to the approximately isocaloric datasets, we estimate *L* at −0.07 (*p* < 0.001; Table S4) from Hu et al.'s [1] data and −0.08 (*p* < 0.001) from Solon‐Biet et al.'s data (Figure 4A,B) [2], confirming the widely reported finding of a partial but statistically robust nonlinear effect of protein leverage in mice. Using the complete datasets with no diet exclusions yields similarly concordant estimates of the strength of protein leverage (Figure 4C,D).

As in Solon‐Biet et al. [2], the data from Hu et al. [1] also indicate regulatory feedback onto the control of food intake associated with carbohydrate/fat (Figure 2; Table S3). The specific contributions from carbohydrate and fat cannot be distinguished in Hu et al. [1], even within the restricted set of diets of similar energy density shown in Figure 3, because these were all of similarly low fat content, leaving too few degrees of freedom for estimating fat‐specific feedback. Solon‐Biet et al. [2] could distinguish all three macronutrients in their design and reported a weaker influence on chronic food intake coming from fat than from carbohydrate, such that compensatory responses to dilution of protein and/or carbohydrate in the diet by fat resulted in increased food and energy intakes.

In summary, we show that even a small amount of leverage by protein (and carbohydrate) can account for the differences in food and energy intakes seen in the studies of Hu et al. [1] and Solon‐Biet et al. [2], without a need to impute hedonics for fat. Therefore, fat can be considered as a high‐energy diluent of protein and carbohydrate, with compensatory feeding for these nutrients driving increased food intake and fat calories coming along for the ride passively.

**FIGURE 5:**
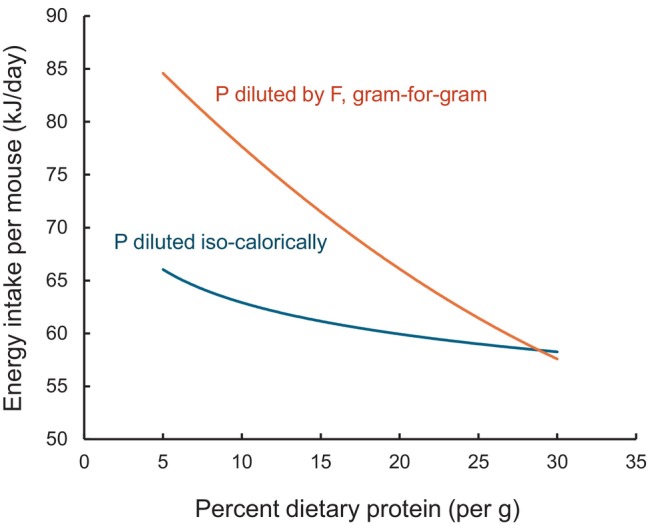
A numerical analysis to demonstrate how protein leverage of strength *L* = −0.07 can account for the increase in energy intake reported by Hu et al. [1]. Based on the data in Figure 4A, food intake (in grams per day) = 3.15 × *p* ^−0.07^, where *p* is the proportion of protein in the diet per gram. Curves are plotted showing estimated caloric intake when protein concentration in the diet is changed by substituting it isocalorically (blue curve) and when protein is substituted by fat on a gram‐for‐gram basis (red curve). See text for detailed explanation.

We understand just how counterintuitive it is that a statistically robust but small level of protein leverage can have such a large effect on energy intake. In order to demonstrate how protein leverage of strength *L* = −0.07 can account for the increase in energy intake reported by Hu et al. [1], we provide a numerical analysis in Figure 5. Based on the data in Figure 4A, we predict that food intake (in grams per day) = 3.15 × *p* ^−0.07^, where *p* is the proportion of protein in the diet per gram. For a diet with 25% protein by weight, a mouse is predicted to eat 3.47 g/day of food. For a diet with 10% protein by weight, a mouse would eat 3.70 g/day of food (i.e., an extra 0.23 g/day). The effects of these increases in food intake on energy intake then depend on the energy density of the diet. The blue line in Figure 5 shows the case in which protein is substituted isocalorically (e.g., by carbohydrate, which is of equivalent energy density to protein, or by a 50:50 mixture of fat and indigestible cellulose, given that fat is twice the energy density of protein), at a total energy density of ~17 kJ/g (i.e., equivalent to the diets in Figure 4A). The intake on the 25% protein diet is predicted to be 59.0 kJ/day, whereas that on the 10% protein diet is 62.9 kJ/day, i.e., an extra 3.9 kJ/day. However, when protein is substituted by fat on a gram‐for‐gram basis (red line in Figure 5), the diets become more energy‐dense, thereby exacerbating the protein leverage effect on energy intake. Assuming a diet that is 25% protein, 5% fats, and 70% carbohydrates per gram gives an energy density of a 17.75‐kJ/g diet, with a mouse predicted to eat 61.6 kJ/day (using the estimated intake of 3.47 g/day). For a diet that is 10% protein, 20% fats, and 70% carbohydrates per gram gives a caloric density of 20.9 kJ/g, with a mouse then predicted to eat 77.3 kJ/day (using the aforementioned estimate of 3.70 g/day of food eaten). This is a now a difference of 15.7 kJ/day; accumulated over the 12 weeks of a dietary experiment, the total difference in energy consumed between a 10% and 25% protein diet would be 1318.8 kJ.

**FIGURE 6:**
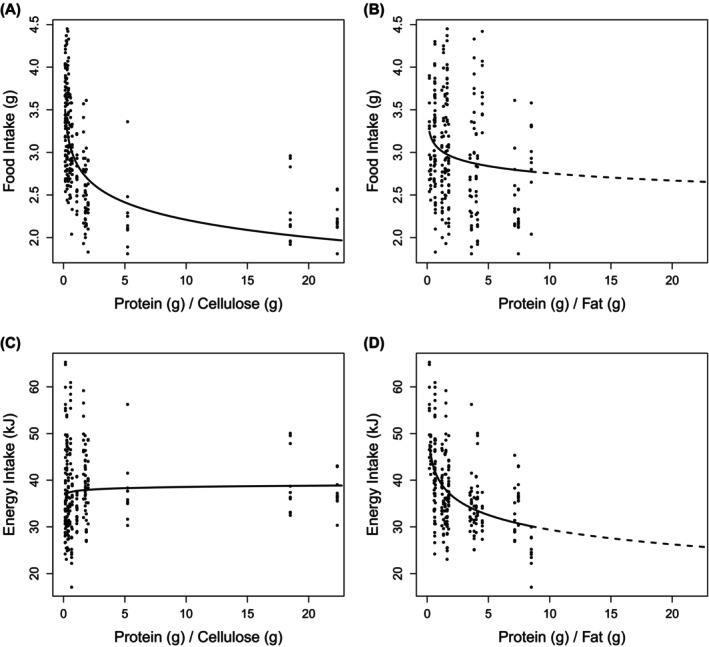
Dilution of dietary protein increases food intake, irrespective of diluent. Effect of the protein to cellulose ratio and the protein to fat ratio on (A,B) food intake (grams) and (C,D) energy intake (kilojoules), based on Solon‐Biet et al. [2]. Lines correspond to fitted values from linear models of intake on the log ratio of protein to nonprotein dietary content. Data are for both sexes, with intakes averaged between 6 and 15 months in C57BL/6 mice. See also Table S6.

Hu et al. [1], by contrast, have argued that elevated energy intake associated with increasing fat content of the diet was due to hedonic factors linked to fat. It is important to note that neither Hu et al. [1] nor Solon‐Biet et al. [2] directly assessed hedonics, as both studies only measured long‐term food intake and thereby hedonics cannot be distinguished from involvement of homeostatic feedback. Nevertheless, it is possible, in the case of the study of Solon‐Biet et al. [2], to test whether increasing fat or decreasing protein content led to increased food intake on low‐protein diets by contrasting food intake when fat or cellulose was used to dilute protein to an equivalent extent by dry mass. Cellulose is nonpalatable and indeed can be considered, in effect, “hypopalatable” because it dilutes more palatable food components in the food matrix. In the data from Solon‐Biet et al. [2], decreasing dietary protein content relative to both cellulose and fat led to statistically significant increases in food intake (Figure 6A,B; Table S6). Thus, dilution of dietary protein robustly and nonlinearly increased food intake, irrespective of the palatability of the diluent. Regardless of whether cellulose or fat was the diluent of protein, food intake increased, but the functional consequences of this increase differed; unlike cellulose, only fat results in increased energy intake because of its caloric content (Figure 6C,D; Table S6).

## DISCUSSION

Changing the concentration of one macronutrient in the diet inevitably affects the ratio with respect to the others, as well as total energy density in the case of manipulation of fats. Understanding the effects of a shift in dietary macronutrient balance and energy density on food and energy intakes requires explicit consideration of the interactions among the macronutrients within a mixture framework. Herein, we have used nutritional geometry to show that the data from the study of Hu et al. [1] are concordant with those from the other most relevant study of macronutrient and energy balancing in mice, i.e., that by Solon‐Biet et al. [2]. In turn, our results demonstrate that the data presented by Hu et al. [1] are concordant with the results of many other published reports of the effect of dietary protein on food intake in mice across strains and sexes [2, 22, 33, 34, 35, 36, 37, 38, 39, 40, 41, 42, 43, 44].

Such studies have shown that protein leverage occurs in mice but is less pronounced than in some other species, being countermanded by compensatory feedback arising from carbohydrates [22] and, to a lesser degree, from fats. Increasing the concentration of fats in the diet leads to elevated long‐term energy intake, not necessarily because fats are highly palatable but rather because they dilute both protein and carbohydrates in the diet, evoking compensatory feeding responses for both nutrients that are sufficient to explain the differences in food and energy intakes across dietary treatments without the need for hedonics. We show that similar effects on food intake can be elicited by dilution of protein and carbohydrates by nonpalatable, indigestible fiber, but the effects on energy intake and thereby adiposity are obviously different to when fats are the diluent.

The chronically increased energy intake that accompanies a fall in dietary protein concentration down to ~10% protein energy is widely reported in rodents to be accompanied by increased energy expenditure, which countermands, but does not prevent, the development of increased adiposity [33, 45, 46], perhaps indicating adaptive diet‐induced thermogenesis [47]. However, with prolonged feeding of mice on a very low‐protein diet (approximately <7% energy from protein), multiple studies have shown lower adiposity [37, 42, 43, 48, 49, 50, 51], which appears to be related to energy expenditure exceeding the increased energy intake induced by low protein [52, 53]. At a protein concentration less than 5%, mice and rats decrease food intake [45, 50]. Such extremely low protein concentrations cannot sustain development, reproduction, and other vital physiological functions that require essential amino acids [54]. Therefore, the compensatory feeding response to protein dilution reaches a breakpoint at very low protein concentrations, presumably reflecting an adaptive response to abandon an untenable diet and seek better foods elsewhere. Although the physiological mechanisms controlling these interlinked processes, and the biological relevance thereof, remain unclear, the peptide hormone fibroblast growth factor 21 (FGF21) is implicated in increasing protein appetite, inhibiting sweet preference, and enhancing energy expenditure under low‐protein, high‐carbohydrate feeding conditions [10, 34, 37, 52, 53, 55, 56, 57].

We are certainly not suggesting that homeostatic feedback is the only mechanism involved in the control of energy intake. Hedonic and homeostatic processes are known to interact, and their relationship changes over time under long‐term restriction to an imbalanced diet. This is evidenced by the commonly reported overriding of immediate palatability effects by longer‐term homeostatic nutritional feedback, which includes state‐dependent modulation of gustatory responses [58, 59, 60]. Therefore, a highly palatable, nutrient‐dense diet will be preferentially selected and initially eaten in larger amounts when provided alone than a less‐palatable, nutrient‐dilute diet, but, as homeostatic feedback kicks in over time, this pattern reverses, giving way to homeostatic correction [61]. Such homeostatic override is even seen when foods are initially aversive rather than palatable [62]. A recent reanalysis of data from Hu et al. [1] by Gao et al. [26] presented food intake data across 12 days following mice being switched to a high‐fat diet. Food intake increased initially relative to baseline but then typically fell back over time, which is indicative of transient orosensory effects being modulated by homeostatic feedback. Likewise, Hu et al. [1] included a series of experiments in which levels of sucrose were altered in the diet and concluded that “the immediate response of the mice to elevated sucrose was to increase intake for several days, but this then returned to the baseline levels, with no long‐term impact on intake or body composition.” This provides another example of hedonic effects being transient.

Relatedly, we are also not saying that acute palatability or food texture effects [63] may not divert an animal away from an optimal macronutrient balance in an environment where the normal food cues involved in homeostatic feeding are disrupted, e.g., when there are excessive food choice options or compositional manipulation [13, 14, 64]. It is in this regard that reconciling alternative interpretations of the control of energy intake in mice becomes of particular importance to understanding human health. In the modern human nutritional environment, the abundance of energy‐dense processed foods specifically designed to be hyperpalatable has diverted the composition of the diet toward an imbalanced state, thereby engaging protein leverage mechanisms with the associated obesity and chronic disease burden [14, 27, 65, 66]. Of particular importance is the fact that the strength of protein leverage in humans is substantially greater than that in mice, with *L* values of between −0.2 and −0.5 being repeatedly reported [14] compared with −0.07 in the mouse. Given its relatively small contribution to energy intake in the human diet (~15%), and thus its large “gearing effect” on the intake of other nutrients, even a small dilution of protein in the food supply by highly palatable, inexpensive, and energy‐dense combinations of fats and carbohydrates has the potential to promote sustained overconsumption of energy [12, 13, 14, 29]. Considering the longer‐term effects of homeostasis, perhaps “hedonic diversion” may be a more appropriate description than “hedonic override” (or “overdrive,” sensu Gao et al. [26]) for mice and possibly also the modern human situation.

## CONFLICT OF INTEREST

The authors declared no conflicts of interest.

## Supporting information


**Data S1.** Supporting Information.

**Table S1.** Diet compositions showing values reported in Hu et al. [1] and derived values calculated from raw ingredients.

## Data Availability

The data that support the findings of this study are available on request from the corresponding author. The data are not publicly available due to privacy or ethical restrictions (https://github.com/AlistairMcNairSenior/Mouse_PL).
