# Supplementary material for: Dietary macronutrient content and energy intake in the mouse: hedonic or homeostatic override?
Source: Obesity (Silver Spring). 2025 Jun 4;33(7):1322–33. doi: 10.1002/oby.24312 (PMC12210109; doi:10.1002/oby.24312)
Supplement: Supplementary file 1 — Data S1. Supporting Information. [file OBY-33-1322-s002.docx]

**Supplemental Information**

**Methods**

Statistical analyses

All statistical analyses were performed in the statistical programming environment *R* version 4.3.1 (31) using data from Solon-Biet et al (2) and Hu et al (1). Multidimensional nutrient surfaces (Figures 2 and S2) for intake were estimated via generalised additive models (GAMs). GAMs were implemented using the ‘gam’ function in the package *mgcv* (68), with the intake of interest (food in grams or energy in kJ) as the response and the content of the diet in terms of different macronutrients (kJ/g protein and non-protein) fitted as a two-dimensional smoothed predictor. Throughout we used GAMs with the family specified as a scaled T distribution (“family = scat()” in *mgcv*), which helps to deal with heavy-tailed distributions. This model reduced heteroscedasticity in the residuals relative to a GAM that assumed a Gaussian distribution.

Linear models (LMs) of percentage protein (Figure 3), and protein ratios (Figure 5) on intakes were implemented using the ‘lm’ function. To make statistical comparisons of effect of dietary protein on intake in the two studies, data were pooled and LMs fitted with an interaction term between the percentage protein in the diet and a categorical predictor denoting the study from which the data came. To estimate the strength of protein leverage (Figure 4) a non-linear least squares model for the equation of food intake = *P*p*^L^* was implemented using the ‘nls’ function, where the outcome was food intake in grams, p was the proportion of the diet that is protein by dry weight, and *P* and *L* are coefficients estimated by the model.

Animals and Husbandry

For data at 24 weeks of age (Figure S3C), 3-week-old male and female C57BL/6 mice were purchased from the Animal Resources Centre (WA, Australia). Mice were housed three per cage in standard approved cages (Techniplast, Varese, Italy) in the Molecular Physiology Unit of the ANZAC Research Institute and maintained at 24-26°C and 44-46% humidity under at 12 h light:dark cycle. To collect food spillage, cages were fit with a custom-designed two-chamber Perspex insert (City West Plastics, NSW, Australia). All protocols were approved the by Sydney Local Health District Animal Welfare Committee (Protocol 2009/003).

**Figure S1, related to Figure 1. Dietary protein content (% of energy) and intake in C57BL/6 mice.**

Mean daily energy (kJ), protein (g) and overall food (g) intake as a function of dietary protein and fat content (% of total energy). Solid points are calculated using the diet compositions given in Hu et al. (1) Table S1, and with missing protein intake data entered and reported as 0 [equivalent to Figure 2A and B in Hu et al. (1)]. Hollow points are calculated using diet compositions derived from the stated raw ingredients, and with missing data entered as NA. Note data points of equivalent % dietary protein have been offset to the left and right to make overlaying points visible. Error bars are SD.

**
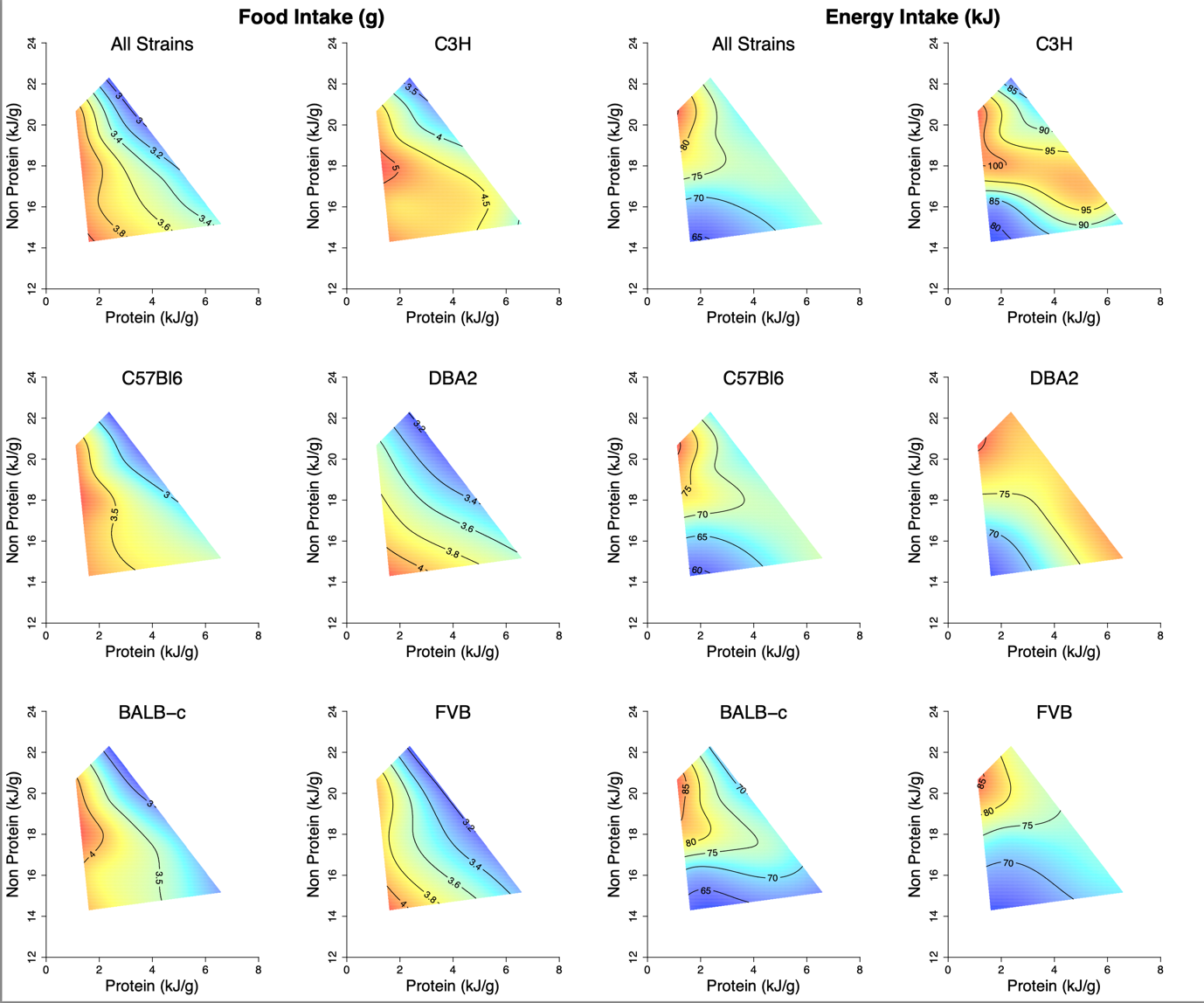
**

**Figure S2, related to Figure 2. Dietary protein and non-protein energy influences on food and energy intake in different strains of mice.** Fitted surfaces from generalised additive models (GAMs) testing for the effects of dietary protein, and non-protein food intake (g) and energy intake (kJ) in different strains of mice in Hu et al. (1). Data from different strains are presented both individually and pooled. Note, for C57Bl/6 animals here, we only considered those diets tested in all strains. See also Table S3. In all panels, surface colours are scaled such that maximal values within the panel are given by red, and minimal values are blue. There was little statistical evidence for strain specificity in effect; for pooled data of all strains, model fit was not significantly improved by the inclusion of a factorial term for the effect of macronutrient content by strain (F-test for analysis of deviance of the two models; food intake (g), df = 18.01, Δ deviance = 7.26, F = 1.11, p = 0.34; energy intake (kJ), df = 18.22, Δ deviance = 3035, F = 1.04, p = 0.41).

**
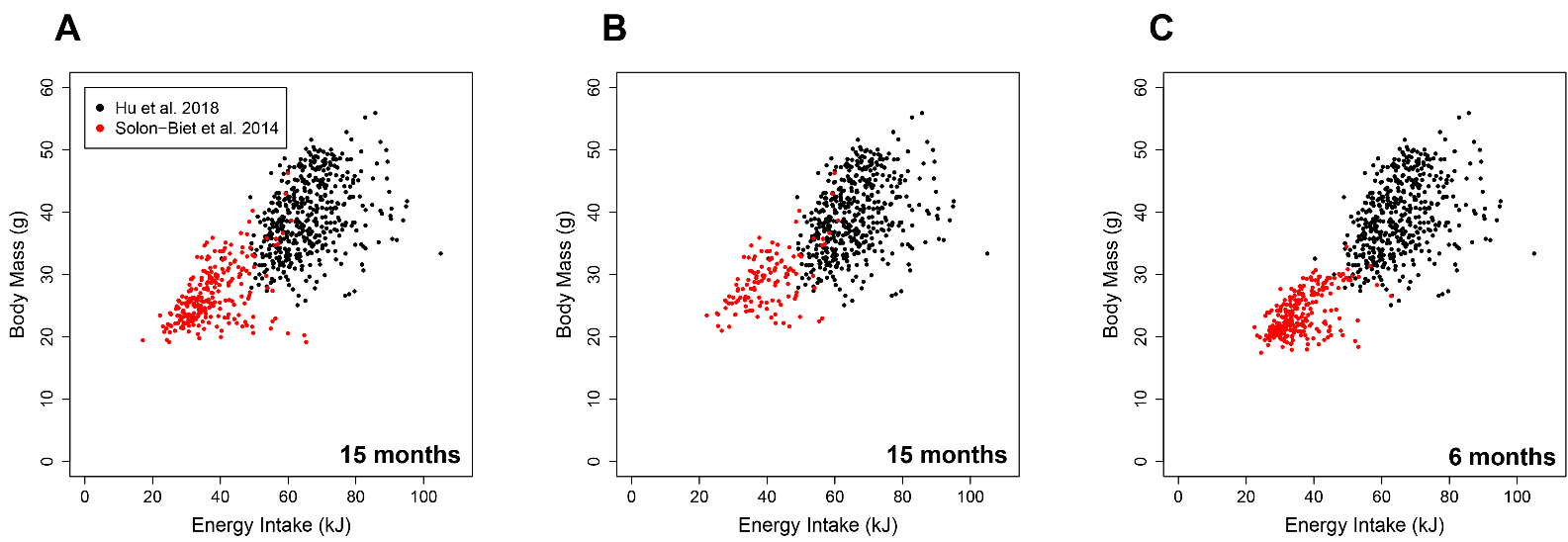
**

**Figure S3. Relationship between energy intake and body mass.** Daily energy intake (kJ) against body mass (g) in Hu et al. (1) (black points) and Solon-Biet et al. (2) (red points). A) Both sexes at 15 months of age, with intake averaged between 6 and 15 months. B) Males at 15 months of age, with intake averaged between 6 and 15 months. C) Body mass for both sexes at 6 months of age, with intake averaged over the first 24 weeks. All data are from C57BL/6 mice.

**Table S1, Related to Figures 1 and S1.** Diet compositions showing values reported in Hu et al. (1) and derived values calculated from raw ingredients. [Attached separately]

**Table S2, Related to Figure 1.** Comparison of study design.

|  | **Hu et al. (1)** | **Solon-Biet et al. (2)** |
| --- | --- | --- |
| **Diet composition** |  |  |
| Protein (%) | 5 - 30% | 5 - 60% |
| Carbohydrate (%) | 8 - 80% | 20 - 75% |
| Fat (%) | 8 - 80% | 20 - 75% |
| Energy (kJ/g) | 16 - 25 | 8 - 17 |
| Protein | Casein | Casein Methionine |
| Carbohydrate | Cornstarch Maltodextrose | Wheatstarch Dextrinized cornstarch Sucrose |
| Fat | Cocoa butter Coconut oil Menhaden oil Palm oil Sunflower oil | Soy oil |
| **Study design** |  |  |
| Sex | Male | Male and Female |
| Strain | C57BL/6 BALB-c C3H DBA2 FVB | C57BL/6 |
| Housing Temp (◦C) | 22-24 | 24-26 |
| Housing density (mice/cage) | 1 | 3 |
| Age of onset (w) | 12 | 3 |
| Age of calculated food intake (w) | 22-24 | 24-60 |

**Table S3, Related to Figure 2 and Figure S3.**

Two dimensional smooth terms from the generalised additive models (GAMs) for effects of dietary protein and non-protein on food intake in terms of mass and energy in the two studies (edf = error degrees of freedom; rdf = reference degrees of freedom). Predictions from response surface models given in Figure 2 and Figure S3.

| **Outcome** | **Study** | **edf** | **rdf** | **F** | **p** |
| --- | --- | --- | --- | --- | --- |
| **Figure 2** |  |  |  |  |  |
| Mass Intake | Hu et al. (1) | 9.749 | 10.752 | 304.947 | <0.001 |
|  | Solon-Biet et al. (2) | 2.001 | 2.002 | 293.572 | <0.001 |
| Energy Intake | Hu et al. (1) | 10.087 | 10.865 | 443.03 | <0.001 |
|  | Solon-Biet et al. (2) | 7.311 | 9.104 | 281.807 | <0.001 |
| **Figure S2** |  |  |  |  |  |
| Mass Intake | All Strains | 8.798 | 10.18 | 264.997 | <0.001 |
|  | C57Bl6 | 8.779 | 10.157 | 163.009 | <0.001 |
|  | BALB-c | 8.304 | 9.836 | 156.428 | <0.001 |
|  | C3H | 8.085 | 9.671 | 82.088 | <0.001 |
|  | DBA2 | 4.704 | 6.163 | 59.997 | <0.001 |
|  | FVB | 5.851 | 7.517 | 48.054 | <0.001 |
| Energy Intake | All Strains | 9.14 | 10.391 | 145.886 | <0.001 |
|  | C57Bl6 | 9.092 | 10.353 | 109.764 | <0.001 |
|  | BALB-c | 8.541 | 10.01 | 97.615 | <0.001 |
|  | C3H | 8.299 | 9.836 | 46.229 | <0.001 |
|  | DBA2 | 5.803 | 7.472 | 37.607 | <0.001 |
|  | FVB | 6.402 | 8.112 | 40.299 | <0.001 |

**Table S4, Related to Figure 3.**

Coefficients (Coef.) from linear model (LMs), estimating (Est.) the effects of percentage (energy) from dietary protein on food intake (grams) and energy intake (kJs) based on data in Hu et al. (1) (15.9 and 18kJ/g diets), and Solon-Biet et al. (2) (17kJ/g diets).

| **Outcome** | **Study** | **Coef.** | **Est.** | **SE** | **t** | **p** |
| --- | --- | --- | --- | --- | --- | --- |
| Mass Intake | Hu et al. | Intercept | 3.831 | 0.055 | 69.49 | <0.001 |
|  |  | % Protein | -0.014 | 0.003 | -5.185 | <0.001 |
|  | Solon-Biet et al. | Intercept | 2.889 | 0.074 | 39.263 | <0.001 |
|  |  | % Protein | -0.012 | 0.003 | -4.925 | <0.001 |
| Energy Intake | Hu et al. | Intercept | 64.227 | 0.923 | 69.551 | <0.001 |
|  |  | % Protein | -0.231 | 0.046 | -4.982 | <0.001 |
|  | Solon-Biet et al. | Intercept | 48.443 | 1.224 | 39.581 | <0.001 |
|  |  | % Protein | -0.207 | 0.042 | -4.949 | <0.001 |

**Table S5, Related to Figure 4.**

Estimated coefficients of *P* and *L* from non-linear least squares model for food intake (g) = *P*p*^L^* where p is the proportion of the food that is protein by dry weight, *P* is the value of intake when p is 1, and *L* is the strength of protein leverage.

| **Dataset** | **Study** | **Coef.** | **Est.** | **SE** | **t** | **p** |
| --- | --- | --- | --- | --- | --- | --- |
| 15.9 – 18kJ/g | Hu et al. | *P* | 3.148 | 0.069 | 45.44 | <0.001 |
|  |  | *L* | -0.068 | 0.011 | -6.056 | <0.001 |
|  | Solon-Biet et al. | *P* | 2.948 | 0.052 | 56.242 | <0.001 |
|  |  | *L* | -0.084 | 0.01 | -8.771 | <0.001 |
| All Data | Hu et al. | *P* | 2.247 | 0.089 | 25.298 | <0.001 |
|  |  | *L* | -0.086 | 0.02 | -4.336 | <0.001 |
|  | Solon-Biet et al. | *P* | 2.534 | 0.082 | 30.945 | <0.001 |
|  |  | *L* | -0.092 | 0.016 | -5.612 | <0.001 |

**Table S6, Related to Figure 5.**

Coefficients (Coef.) from linear model (LMs), estimating (Est.) the effects of log natural (ln) ratio of protein (P) to cellulose and fat (F) in the diet (g) on mass intake (g) and energy intake (kJ) based on data from Solon-Biet et al. (2). Model predictions were back-transformed for plotting in Figure 5.

| **Outcome** | **Ratio** | **Coef.** | **Est.** | **SE** | **t** | **p** |
| --- | --- | --- | --- | --- | --- | --- |
| Mass Intake | P / cellulose | Intercept | 2.888 | 0.03 | 95.522 | <0.001 |
|  |  | ln Ratio | -0.295 | 0.022 | -13.657 | <0.001 |
|  | P / F | Intercept | 3.036 | 0.043 | 70.522 | <0.001 |
|  |  | ln Ratio | -0.122 | 0.039 | -3.155 | <0.01 |
| Energy Intake | P / cellulose | Intercept | 37.683 | 0.55 | 68.486 | <0.001 |
|  |  | ln Ratio | 0.379 | 0.393 | 0.965 | 0.34 |
|  | P / F | Intercept | 39.857 | 0.513 | 77.744 | <0.001 |
|  |  | ln Ratio | -4.543 | 0.461 | -9.848 | <0.001 |
